# Cold Atmospheric Plasma as a Potential Disease-Modifying Therapy for Osteoarthritis

**DOI:** 10.3390/biomedicines14071494

**Published:** 2026-07-01

**Authors:** Vinay Kumar, Fiona O’Neill, Emma J. Murphy, Declan M. Devine, Liam O’Neill, Niamh Fahy

**Affiliations:** 1 Bioengineering Organ-on-Chip Research Group, Centre for Applied Bioscience Research, Moylish Campus, Technological University of the Shannon, V94 EC5T Limerick, Ireland; vinay.kumar@tus.ie (V.K.); emma.murphy@tus.ie (E.J.M.); 2 Department of Applied Science, Moylish Campus, Technological University of the Shannon, V94 EC5T Limerick, Ireland; 3 TheraDep Ltd., QUESTUM Acceleration Centre, Clonmel, E91 V329 Co. Tipperary, Ireland; fionamoneill@theradep.com (F.O.); liamoneill@theradep.com (L.O.); 4 PRISM Research Institute, Athlone Campus, Technological University of the Shannon, Athlone, N37 HD68 Co. Westmeath, Ireland; declan.devine@tus.ie

**Keywords:** cartilage, cold atmospheric plasma, inflammation, osteoarthritis, oxidative stress, reactive oxygen and nitrogen species, synovial membrane

## Abstract

Osteoarthritis (OA) is a disabling joint disease characterised by cartilage degradation, synovial inflammation, and subchondral bone remodelling. Furthermore, catabolic inflammatory processes as well as dysregulated cellular signalling and oxidative stress are central to OA pathogenesis. Despite its growing global burden, currently available therapies primarily provide symptomatic relief and fail to target underlying molecular mechanisms and halt disease progression. Cold atmospheric plasma (CAP), a partially ionised, non-thermal gas that generates controlled reactive oxygen and nitrogen species (RONS), has emerged as a promising therapeutic modality capable of modulating redox-sensitive signalling pathways. CAP has demonstrated the capacity to suppress pro-inflammatory cytokine expression, enhance antioxidant defence mechanisms, influence macrophage polarisation, and stimulate tissue repair processes in rheumatoid arthritis, diabetic and dermal wound healing models. However, its potential as a disease-modifying therapy for the treatment of OA is not yet fully understood and warrants further experimental investigation. This review explores current pre-clinical evidence from different disease models, which may have implications for the potential application of CAP as a therapeutic intervention for OA, either as a disease-modifying therapy or as an adjuvant therapy for intra-articular drug delivery. Furthermore, key translational challenges including plasma parameter standardisation, interactions with synovial fluid and optimisation of joint-specific delivery strategies are discussed, identifying gaps that require further experimental investigation. Collectively, the findings of this review highlight CAP as a promising multimodal therapy with translational potential for the treatment of OA warranting further experimental validation and may open innovative avenues for future research.

## 1. Introduction

Osteoarthritis (OA) is a chronic degenerative disease of synovial joints and a leading cause of pain and functional disability worldwide [1]. In 2020, an estimated 595 million people were living with OA, with knee OA representing the most common clinical presentation [2,3]. OA development is influenced by multiple risk factors including ageing, sex, obesity, metabolic disorders, mechanical overloading, trauma, and genetic susceptibility [4,5,6,7]. Pathologically, OA affects the entire joint organ, involving cartilage, subchondral bone, synovium, ligaments, joint capsule and peri-articular muscles [8].

Despite major advances in understanding OA pathophysiology, currently approved interventions remain largely symptom-focused rather than disease-modifying. Oral non-steroidal anti-inflammatory drugs (NSAIDs) and intra-articular injections (e.g., corticosteroids, hyaluronic acid) provide analgesia and short-term anti-inflammatory relief, but do not reliably prevent structural progression [9,10,11,12,13]. For end-stage disease, surgical interventions remain the standard care [14]. However, the limited lifespan of joint prostheses as well as rehabilitation burden and risk of complications make it an imperfect solution, particularly for younger patients [15,16].

Regenerative approaches, including orthobiologics (e.g., platelet-rich plasma, mesenchymal stem cells) and cartilage repair techniques (e.g., autologous chondrocyte implantation) offer short- to mid-term relief but are limited by procedural invasiveness, lack of standardised protocols, the formation of mechanically inferior fibrocartilage, and the catabolic intra-articular environment delaying tissue regeneration [17,18,19,20]. This highlights a need for novel minimally invasive approaches capable not only of slowing structural decline within an OA joint, but of actively reversing the underlying pathological processes driving it. The development of novel disease-modifying therapies for the treatment of OA is an area of active investigation, with emerging strategies focused on pharmacological interventions targeting underlying biological mechanisms including dysregulated cellular signalling cascades and inflammatory processes [21,22,23]. Furthermore, advanced diagnostic methods for the early detection and monitoring of biomechanical and biochemical changes to articular cartilage are under development, facilitating the assessment and potential treatment of early disease processes [24]. Cold atmospheric plasma (CAP) is an additional novel candidate which may hold translational promise for OA. CAP is a non-thermal, partially ionised gas generated at atmospheric pressure and produces reactive oxygen and nitrogen species (RONS), which can diffuse and interact with biological tissues [25]. Although CAP is currently under-investigated in OA-specific models, CAP exposure has demonstrated downregulation of pro-inflammatory cytokines (Interleukin (IL)-1β, Tumour Necrosis Factor (TNF)-α, modulation of oxidative stress through redox-sensitive mechanisms [26,27] and facilitation of regenerative processes [28,29,30] across various different experimental models, raising the possibility that CAP may have the potential to address several pathological hallmarks of OA simultaneously.

This narrative review summarises the key pathological pathways underlying OA progression and critically evaluates CAP’s reported anti-inflammatory, antioxidant, and regenerative effects across different in vitro and in vivo models. The literature was identified through Scopus, Google Scholar and PubMed using a combination of the following keywords: ‘cold atmospheric plasma’, ‘CAP’, ‘osteoarthritis’, ‘rheumatoid arthritis’, ‘wound healing’, ‘inflammation’, ‘oxidative stress’, ‘plasma medicine’, ‘synovium’, ‘reactive oxygen and nitrogen species’, ‘synoviocytes’, ‘cartilage’, ‘non-thermal plasma’, ‘macrophage polarisation’, ‘cytotoxicity’, ‘genotoxicity’ and ‘regeneration’. Studies were selected based on relevance to OA pathogenesis, tissue regeneration, oxidative stress and inflammatory modulation. Based on these selected studies, many of which are from non-OA-specific models, we hypothesise that CAP may hold promise as a disease-modifying intervention for OA through RONS-mediated immunomodulation, restoration of mitochondrial redox homeostasis, and stimulation of tissue repair. Finally, the potential of CAP as an adjuvant therapy for OA and current translational challenges are discussed, including parameter standardisation, interactions with synovial fluid, and optimisation of knee-joint-specific delivery and dosing strategies.

## 2. OA Pathogenesis

**Figure 1:**
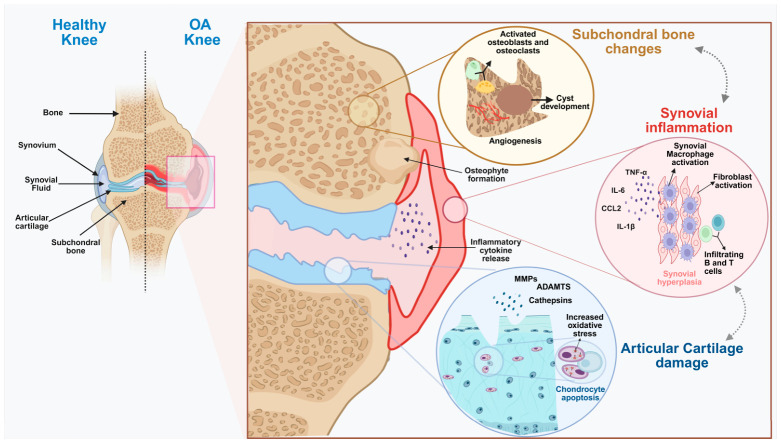
Comparison of a healthy knee joint with an osteoarthritic (OA) knee, highlighting key pathological changes involved in OA progression. ADAMTS: A Disintegrin and Metalloproteinase with Thrombospondin Motifs, CCL2: Chemokine (C-C motif) Ligand 2, IL-1β: Interleukin-1 beta, IL-6: Interleukin-6, MMPs: Matrix Metalloproteinases, TNF-α: Tumour Necrosis Factor-alpha. Created in BioRender. Fahy, N. (2026) https://BioRender.com/6smwt6d, accessed on 27 May 2026.

OA represents a complex biomechanical failure of the entire joint organ which arises from cartilage degradation, synovial inflammation, osteophyte formation, subchondral bone remodelling, and dysregulated cellular redox homeostasis. Each process amplifies the others, collectively destabilising joint homeostasis (Figure 1).

### 2.1. Cartilage Degeneration in OA

Abnormal biomechanical stress and trauma expose superficial chondrocytes to catabolic and immune mediators present within the synovial fluid [31]. In response to this environment, chondrocytes lose their characteristic phenotype, undergo hypertrophic differentiation, and upregulate the production of pro-inflammatory cytokines and destructive enzymes such as proteoglycans aggrecanases (A Disintegrin and Metalloproteinase with Thrombospondin Motifs (ADAMTS)-4 and ADAMTS-5), stromelysins (Matrix Metalloproteinases (MMP)-3), and cathepsins (B, K, and L). These collectively drive the progressive loss of proteoglycans from the cartilage extracellular matrix (ECM) [24,31].

The loss of proteoglycans exposes the underlying collagen network, which is subsequently cleaved by collagenases such as MMP-13 into smaller fragments [32]. These fragments are further processed by gelatinases (MMP-2 and MMP-9) to induce irreversible collagen degradation [33,34]. Additionally, these fragments function as damage-associated molecular patterns (DAMPs), stimulating Toll-like receptors (TLRs) and the receptor for advanced glycation end-products (RAGE) signalling pathways, thereby worsening inflammation and propagating ECM degradation [6,35]. ECM fragments released into the synovial fluid thereby maintain a cycle of inflammation and matrix degradation through continual induction of cytokine expression, including IL-1β and TNF-α [36].

### 2.2. Synovial Inflammation

Under physiological conditions, synovial fluid, the synovial lining (synovium), and resident macrophage populations collectively maintain joint homeostasis. During OA, cartilage-derived DAMPs activate synovium-resident fibroblast-like synoviocytes (FLS) and synovial macrophages via pattern recognition receptors (PRRs) such as TLRs, disrupting the immunological balance of the joint [37]. Furthermore, altered trace element levels including iron and copper have been reported to associate with OA pathogenesis, having the capacity to mediate inflammatory processes and cellular oxidative stress [38,39]. Persistent stimulation promotes macrophage polarisation toward a pro-inflammatory M1 phenotype which is consistently observed in experimental OA models [40,41,42]. While macrophages of the M2 phenotype are known to promote tissue repair, anti-inflammatory responses and the phagocytic clearance of apoptotic cells, their capacity to counter the catabolic environment in OA remains unclear [43,44,45].

Activated M1 macrophages and FLS drive an inflammatory immune response through the secretion of pro-inflammatory cytokines and chemokines including TNF-α, IL-1β, IL-6, IL-12, IL-23, together with RONS, amplifying inflammatory signalling and altering synovial fluid composition [43,44,45,46]. M1 macrophages also establish a catabolic network with chondrocytes, mediated by extracellular vesicles and inflammatory cytokines, that directly induces chondrocyte apoptosis and suppresses matrix synthesis, worsening cartilage degradation [45,47]. At the joint margins, synovial macrophages mediate osteophyte formation in OA through transforming growth factor-beta (TGF-β) driven Bone Morphogenetic Protein (BMP)-2/BMP-4 signalling [48,49]. Simultaneously, FLS and macrophage-derived chemokines (C-C motif Ligand (CCL)2 and CCL3) promote synovial infiltration of monocytes and lymphocytes, driving expansion of the synovial lining (hyperplasia), stromal fibrosis, and angiogenesis, compounding structural deterioration [37,50].

### 2.3. Subchondral Bone Changes

Abnormal mechanical loading and inflammatory signalling drive substantial remodelling within subchondral bone during OA progression. Early disease stages are characterised by increased expression of receptor activator of nuclear factor kappa-B ligand (RANKL) in osteocytes, which promotes osteoclastogenesis and excessive bone resorption, leading to osteocyte apoptosis and microdamage accumulation [51].

As disease progresses, bone remodelling shifts towards osteoblast-mediated bone formation through the activation of Wnt signalling pathways and suppression of sclerostin expression [51,52]. Simultaneously, TGF-β1, upregulated from osteoclast resorption sites, recruits osteoprogenitors and promotes osteoid formation, leading to microfractures in subchondral bone and the osteochondral junction, increased bone turnover, sclerosis, and the development of subchondral bone cysts and bone marrow lesions [51,53,54,55].

This remodelling is compounded by biochemical cross talk with the inflamed synovium [45]. Pro-inflammatory cytokines and factors such as TGF-β1 and vascular endothelial growth factor (VEGF) secreted by osteoclasts, preosteoclasts, and infiltrating polarised macrophages promote pathological neovascularisation at the osteochondral junction, facilitating infiltration of inflammatory mediators into subchondral bone and accelerating cartilage degeneration [45,51,53,56].

### 2.4. Cellular Oxidative Stress and Mitochondrial Dysfunction

Redox imbalance is increasingly recognised as central to OA pathogenesis. Mechanical stress and metabolic disturbances disrupt redox homeostasis, causing mitochondrial dysfunction characterised by impaired adenosine triphosphate (ATP) production, mitochondrial DNA mutations, and defective autophagy, alongside the upregulation of pro-oxidant enzymes and suppression of antioxidant defences [57,58,59,60,61,62,63]. The resulting excess of reactive oxygen species (ROS) activates redox-sensitive signalling pathways such as NF-κB and mitogen-activated protein kinase (MAPK), upregulating the expression of inflammatory cytokines and matrix-degrading enzymes [64,65]. Additionally, downregulation of selenium metabolism impairs selenium-dependent antioxidant capacity, contributing to oxidative DNA damage, mitochondrial dysfunction, and chondrocyte apoptosis [66]. Impaired activation of the nuclear factor erythroid 2-related factor 2 (NRF2) pathway further compromises cellular antioxidant response to counteract inflammasomes, such as NOD-like receptor pyrin domain-containing 3 (NLRP3), leading to upregulation of inflammatory cytokines and further joint damage [67,68].

### 2.5. OA: An Interconnected Whole-Joint Disease

The pathological processes described above act concurrently within the joint. Biomechanical degradation of hyaline cartilage releases DAMPs that drive synovial macrophage M1 polarisation, excessive ROS production, and chondrocyte apoptosis, while simultaneously disrupting osteochondral remodelling through RANKL-mediated osteoclastogenesis and aberrant Wnt signalling (Figure 1). These events form a self-reinforcing cycle of cartilage degradation, synovial inflammation, and pathological bone remodelling, compounded by mitochondrial dysfunction and redox imbalance. Novel therapeutic interventions that operate across oxidative, immune, and osteochondral pathways simultaneously are required. Plasma medicine, particularly through the application of CAP, offers a promising multifunctional approach by harnessing RONS-mediated mechanisms to target these interconnected axes.

## 3. Plasma Medicine: Principles and Therapeutic Mechanisms

Plasma is a partially ionised gas composed of electrons, ions, neutral excited species, and free radicals that collectively behave in a quasi-neutral state [69]. When generated at atmospheric pressure while maintaining near-room temperature, it is referred to as CAP. This allows CAP to be applied directly to biological tissues without inducing thermal cytotoxicity. The most common methods for generating plasma are dielectric barrier discharge (DBD) and atmospheric pressure plasma jet (APPJ) configurations [70]. Regardless of device configuration, CAP produces RONS and electromagnetic radiation [71]. Factors such as power, gas type, exposure mode, duration, equipment configuration, and gas flow rate influence the specific concentration and ratio of RONS generated [72,73].

The biological activity of CAP is primarily mediated through the controlled delivery of RONS, which interact with cellular membranes and intracellular signalling [74]. Through RONS-mediated interactions, CAP has shown potential to inhibit tumour growth, provide antimicrobial activity, and enhance regenerative processes in wound healing, cancer therapy and dental treatments [75,76,77]. In addition to direct CAP treatment of biological tissues, indirect CAP treatment of the environment surrounding cells and tissues is a further area of extensive research, including the surface modification of tissue engineering scaffolds. CAP surface modification of scaffolds has the capacity to improve stem cell attachment and promote osteogenic and chondrogenic differentiation of mesenchymal stem cells [78,79], highlighting its potential also in regenerative medicine applications.

## 4. Therapeutic Relevance of CAP for OA

The biomedical utility of CAP depends on its capacity to deliver controlled and localised RONS capable of modulating redox-sensitive signalling pathways. Current evidence drawn from studies investigating the effect of CAP towards human monocyte, keratinocyte and gingival fibroblast cell lines, as well as primary human rheumatoid arthritis fibroblast-like synoviocytes (RA-FLS), OA fibroblast-like synoviocytes, dermal fibroblasts and keratinocytes in vitro (Table A1) and its application in dermal, diabetic and rheumatoid arthritis (RA) in vivo models (Table A2), suggest that CAP can modulate inflammation, oxidative stress, and tissue regeneration processes. Although these findings provide mechanistic rationale for potential application of CAP in OA, it is important to note that CAP treatment has not been investigated in pre-clinical OA-specific models to date and the proposed therapeutic relevance of CAP to OA remains inferential and requires further experimental validation.

### 4.1. Anti-Inflammatory Activity of CAP

Inflammation drives OA pathogenesis through the production of cytokines, chemokines and matrix-degrading enzymes. Although evidence from OA-specific models is currently lacking, reports from RA models suggests that CAP can modulate inflammatory processes within synovial tissue. In a rat model of adjuvant-induced RA, intra-articular CAP delivery reduced synovial hyperplasia and inflammatory infiltration [80,81]. CAP may also shift macrophage polarisation toward an anti-inflammatory phenotype. A study by Crestale et al. using human primary monocyte-derived macrophages reported decreased M1 surface marker expression and increased M2 subset markers following CAP exposure, indicating CAP’s capacity to alter macrophage phenotype [82]. Furthermore, CAP has been reported to reduce synovial fibroblast-driven inflammation by suppressing NF-κB activity (decreased RelA mRNA levels) and IL-6 secretion in both basal and TNF-α/lipopolysaccharide (LPS)-stimulated primary human RA-FLS cultures in vitro, as well as reducing RANKL expression in unstimulated cells Additionally, MMP-3 expression was reduced in resting cells, suggesting a capacity to limit matrix-degrading pathways [83]. In a separate in vitro study conducted using unstimulated OA-derived fibroblast-like synoviocytes (OA-FLS) cultures, a significant reduction in IL-6 secretion was reported. However, CAP treatment did not significantly alter NF-κB (RelA), RANKL, or MMP-3 expression [84]. In both studies, RA-FLS or OA-FLS were similarly treated with direct application of Argon-based CAP using a plasma jet device at a fixed distance of 1.5 cm from the surface of the culture medium for 90 s. Notably, RA patients were reported to have higher baseline levels of systemic inflammation markers (erythrocyte sedimentation rate and C-reactive protein) compared to OA patients. The reported differences in cell behaviour may likely reflect the lower inflammatory baseline and distinct cellular phenotype of OA-FLS relative to RA-FLS. A more expansive molecular analysis as well as optimisation of CAP treatment protocols may be required to elucidate the potential of CAP for modulation of inflammation in OA.

Beyond joint-resident cells and tissues, CAP has shown anti-inflammatory activity across multiple non-OA-specific experimental models (Table A1 and Table A2). CAP treatment of stimulated human monocyte and keratinocyte cell lines in vitro has been reported to downregulate the expression of pro-inflammatory cytokines including IL-1β, IL-6, and TNF-α, while activating antioxidant defence pathways such as NRF2 signalling [85,86]. These findings were translated in vivo across multiple experimental models, including murine skin inflammation and diabetic wound models, where CAP treatment reduced the expression of IL-6 and TNF-α, and suppressed Janus kinase/signal transducer and activator of transcription (JAK/STAT) signalling, accompanied by reduced leukocyte and macrophage infiltration [86,87,88,89]. In a clinical trial involving patients with diabetic foot ulcers, the downregulation of IL-1, IL-8, interferon (IFN)-γ, and TNF-α expression and accelerated tissue repair was reported following CAP treatment [90].

### 4.2. Effect of CAP on Oxidative Stress and Mitochondrial Modulation

Disruption of cellular redox balance and mitochondrial dysfunction are central contributors to OA progression. Although the application of CAP in OA-specific models requires further research, current evidence from non-OA-specific models suggests that cellular exposure to CAP may have the potential to restore both pathways. In an erastin-stimulated human gingival fibroblast in vitro model, CAP downregulated inducible nitric oxide synthase (iNOS), cyclooxygenase (COX)-2, IL-1β, TNF-α, and IL-6 while enhancing antioxidant responses through increased glutathione peroxidase 4 (GPX4) expression, with associated reductions in intracellular oxidative stress and improved mitochondrial membrane potential [91]. In vivo studies utilising non-OA-specific models further support the antioxidant capacity of CAP (Table A2). In a streptozotocin (STZ) and nicotinamide-induced diabetes mouse model, CAP treatment of mice was shown to reduce biomarkers of oxidative damage such as malondialdehyde (MDA), advanced oxidation protein products (AOPP), and oxidised low-density lipoprotein (oxLDL), while increasing antioxidant enzyme activity including superoxide dismutase (SOD), catalase and glutathione [88]. In a rat model of adjuvant-induced RA, CAP enhanced endogenous antioxidant defence in synovial tissues, accompanied by reduced lipid peroxidation and oxidative damage [80].

Cellular responses to CAP may vary across cell different cell types, and current evidence on the potential of CAP to modulate oxidative stress pathways and mitochondrial dysfunction was well as inflammation in OA is limited. CAP treatment of unstimulated OA-FLS was reported to not significantly alter levels of the lipid peroxidation marker MDA, suggesting that the oxidative stress-modulating effects of CAP may depend on the cellular context and baseline inflammatory state [84]. Furthermore, in vitro culture expanded OA-FLS cells may not reflect the multifaceted intra-articular environment in vivo, and additional experimental research is required to further explore the potential of CAP for OA.

### 4.3. Effect of CAP on Tissue Regeneration Processes

In addition to suppressing inflammatory and oxidative pathways, CAP has demonstrated regenerative effects in non-OA-specific models that may be relevant for restoring tissue integrity in degenerative conditions. However, its potential for OA requires further experimental research. Various in vitro and in vivo studies report upregulation of pro-chondrogenic and pro-healing growth factors, VEGF and TGF-β, and stimulation of collagen type I in skin wound models with STZ-induced diabetic and db/db mice, as well as wild-type mice, suggesting improved extracellular matrix synthesis and tissue repair [92,93,94,95]. In clinical trials of patients with chronic wounds, CAP has been reported to accelerate tissue repair rates, with upregulation of FGF-2 and VEGF-A alongside reduction in inflammatory cytokines [96,97]. While demonstrated primarily in wound healing models and clinical trials, CAP’s capacity to promote tissue regeneration processes, including TGF-β signalling and collagen matrix synthesis, raises the possibility of application in cartilage repair and mitigation of degenerative processes in OA.

### 4.4. Hypothesised Therapeutic Mechanism of Action of CAP in OA: Potential to Modulate Articular Cartilage Damage and Synovial Inflammation?

**Figure 2:**
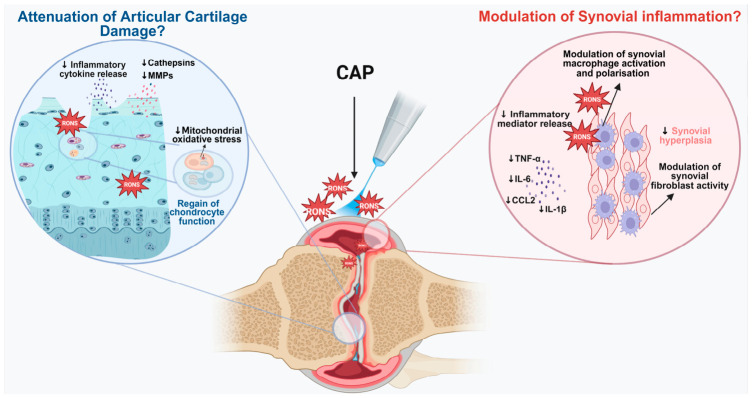
Hypothesised therapeutic mechanisms of action of CAP in an OA knee requiring further validation in OA-specific experimental models. CAP: cold atmospheric plasma, CCL2: Chemokine (C-C motif) Ligand 2, IL-1β: Interleukin-1 beta, IL-6: Interleukin-6, MMPs: Matrix Metalloproteinases, OA: osteoarthritis, RONS: reactive oxygen and nitrogen species, TNF-α: Tumour Necrosis Factor-alpha. Created in BioRender. Fahy, N. (2026) https://BioRender.com/ytuou1n, accessed on 27 May 2026.

CAP may have the potential to influence OA progression through several interconnected biological mechanisms; however, further experimental research in OA-specific models is required to elucidate its therapeutic capacity. Findings extrapolated from current pre-clinical investigations in non-OA-specific models suggest that CAP may have the capacity to suppress the expression of key pro-inflammatory cytokines including IL-1β, TNF-α, and IL-6, while shifting macrophage polarisation away from the pro-inflammatory M1 phenotype. Furthermore, controlled RONS delivery may activate antioxidant pathways, improve mitochondrial function and reduce oxidative damage within joint tissues. CAP has also been reported in non-OA-specific models to stimulate growth factor signalling and ECM synthesis, supporting tissue repair. However, the capacity of CAP to attenuate cartilage damage and modulate synovial inflammation in an OA joint is currently unclear and warrants additional experimental research. Figure 2 represents a hypothesised therapeutic mechanism of action of CAP inferred from existing studies of non-OA models. However, these hypothesised mechanisms required validation and further research in OA-specific models.

## 5. CAP as a Therapeutic Adjuvant

Although CAP has shown standalone therapeutic potential in various pre-clinical and clinical disease models, it can also function as an adjuvant therapy. Investigations on the application of CAP as a therapeutic adjuvant for intra-articular drug delivery to synovial joint tissues are currently limited, but studies have reported the use of HeLa monolayer, blood–brain barrier, dermal wound models, and micromotor systems (Table 1). CAP exposure transiently increases the cell membrane permeability of HeLa cells and mouse immortalised brain endothelial cells, thereby enhancing intracellular uptake of therapeutic molecules [98,99]. This property has been explored to improve drug delivery efficiency by facilitating transport of small molecules and macromolecules across cellular membranes. Additionally, CAP has demonstrated synergistic capabilities, enhancing drug release from plasma-activated carriers and increasing absorption in skin [100,101], and has been proposed as a drug deposition platform capable of delivering therapeutic agents directly onto biological surfaces [30].

In the context of OA, CAP may therefore complement existing pharmacological therapies or emerging disease-modifying osteoarthritis drugs by increasing cellular permeability and enhancing local drug penetration, potentially improving intra-articular delivery while reducing systemic exposure. CAP may also act synergistically with regenerative therapies, including platelet-rich plasma, mesenchymal stem cell therapies, and cartilage repair procedures such as autologous chondrocyte implantation (ACI) or matrix-assisted ACI, to improve therapeutic outcomes. However, these applications are hypothetical and their translatability to OA remain unestablished. Further pre-clinical studies are required to validate these proposed mechanisms.

## 6. Challenges and Future Directions

Before CAP can be translated into a clinically viable intra-articular therapy for OA, several challenges require attention.

### 6.1. CAP Parameter Standardisation

CAP systems vary widely in device configuration (e.g., DBD, APPJ, or hybrid systems) and operational parameters (e.g., voltages, exposure times, gas types) and these variables significantly influence the resulting RONS concentrations [72,73]. Effects also vary across tissue types [80,84,91]. Standardised treatment protocols and dosing will therefore be essential for reproducibility and clinical translation.

### 6.2. Synovial Fluid Barrier, RONS Dosimetry, Safety Considerations and Intra-Articular Delivery Strategies

Major challenges for direct intra-articular CAP therapy include the biochemical barrier presented by synovial fluid, potential dose-dependent cytotoxicity, the effects of RONS on chondrocytes, synoviocytes, and the extracellular matrix, the risk of excessive oxidative stress, and the possible consequences of repeated intra-articular CAP applications.

Synovial fluid (SF) is composed of high concentrations of hyaluronic acid (HA), phospholipids, and proteins. HA and SF have been previously reported to scavenge excessive ROS, suggesting that SF may quench CAP-generated RONS before they reach target tissues within the joint [102]. Furthermore, the presence of excessive ROS can also degrade HA, reducing its molecular weight, impacting viscosity and lubrication of the joint [103,104]. Such changes in SF viscosity may in turn alter the diffusion kinetics of RONS within the joint cavity. Additionally, phospholipids and proteins present in SF may chemically interact with CAP-generated RONS and modulate its effects, although direct evidence in CAP-treated SF is currently absent [105,106]. The primary therapeutic mode of action of CAP in the joint is therefore unlikely to be direct plasma-to-tissue contact, but rather plasma-activated synovial fluid (PASF), where a proportion of RONS will survive quenching and a proportion will be retained in SF before interacting with joint tissues. Future studies should investigate the diffusion kinetics, stability, and biological effects of reactive species within PASF to confirm that therapeutically relevant concentrations reach target tissues without inducing SF modification and cytotoxic effects.

A critical safety concern is dose-dependent cytotoxic effects of CAP-derived RONS. In in vitro studies with cell lines including glioma, osteosarcoma, fibroblasts and mesenchymal stem cells, prolonged exposure to CAP has been shown to reduce cell viability and induce apoptosis in a dose-dependent manner, whereas short exposure time may enhance cell proliferation [107,108,109,110]. Genotoxic and mutagenic effects have also been reported in malignant and non-malignant cells [108,110,111]. Conversely, previous studies have reported that moderate treatment with an argon plasma jet does not increase genotoxicity [112,113], and no severe side effects of CAP treatment were observed in a clinical oncology study [114]. As described in Section 2.4, the OA joint is characterised by elevated oxidative stress and impaired antioxidant defences. CAP-generated RONS may therefore induce excessive oxidative stress and lead to cytotoxicity and genotoxicity in chondrocytes, synoviocytes, and subchondral bone cells, and may additionally compromise ECM integrity by the degradation of collagen and proteoglycans. However, cytotoxicity and genotoxicity data are currently unavailable for relevant OA models, and the differential sensitivity of joint-resident cell types to CAP-derived RONS may further complicate dose optimisation. These findings highlight the need for effective dosing parameters based on individual cell types and an understanding of integrated tissue behaviour before CAP can be translated to intra-articular application. Even after defining a safe dose, the consequences of repeated exposure remain unknown and may require long-term studies to understand cumulative RONS effects on joint tissue.

The clinical translation of CAP for OA will also require development of safe, reproducible and joint-specific delivery strategies. Direct arthroscopic or catheter-based procedures could allow localised treatment of synovial tissue and cartilage, as demonstrated by Ding et al. in a pre-clinical RA study where CAP was delivered directly to the joint cavity [80]. In a clinical setting, such approaches will require strict controls for gas exposure, treatment uniformity, and validated sterility and safety protocols. Plasma-activated fluid and hydrogels may offer a more practical approach. For example, plasma-activated saline has demonstrated biological activity and synergistic efficacy with pharmaceutical agents in an infection model [115], while hydrogels have been explored as a platform for controlled release of reactive species [116]. Further investigations are required to evaluate such strategies in OA-specific models, and to evaluate optimal plasma-activated fluid compositions, storage conditions and half-life, as well as SF and joint tissue interactions.

### 6.3. Therapeutic Window for Intra-Articular Application

The therapeutic efficacy of CAP for the treatment of OA is likely to depend on disease stage. CAP-based intervention may hold most promise during early to moderate OA, when modulation of inflammation, oxidative stress, and cellular homeostasis may have the capacity to slow cartilage degradation or preserve remaining cartilage tissue. In advanced OA with extensive cartilage loss, CAP-based interventions may be beneficial in assisting other regenerative cartilage repair procedures. However, further research is required to determine potential therapeutic windows using OA-specific pre-clinical models.

### 6.4. Current Lack of OA-Specific Models Investigating CAP Therapy

The majority of in vivo CAP research to date has utilised dermatological or diabetic wound models (Table A2). While these demonstrate redox modulation and tissue repair, they do not replicate the complex biomechanical and immunological environment of the OA joint. Notably, intra-articular CAP delivery has been investigated in a rat model of adjuvant-induced RA [80], demonstrating proof-of-concept for joint-specific application. However, evidence in OA pre-clinical models remains absent, representing a critical gap in the translational pathway. Validating CAP’s intra-articular efficacy in OA would therefore require advanced platforms including joint-on-chip technologies that better recapitulate OA-specific pathology.

## 7. Conclusions

Current evidence extrapolated from pre-clinical and clinical investigations in non-OA-specific models suggests that CAP may represent a minimally invasive approach with disease-modifying potential for OA through its inflammatory, oxidative, and regenerative properties. Further research is required to address key challenges including the optimisation of plasma parameters and characterisation of RONS behaviour within synovial fluid, as well as validation of therapeutic benefit in OA-specific experimental models. Whether used alone or alongside existing pharmacological and regenerative therapies, CAP warrants further investigation as a disease-modifying intervention for OA.

## Figures and Tables

**Table 1:** Therapeutic Effects of CAP in Drug Delivery Applications in non-OA-specific models.

Therapeutic Role of CAP	Experimental Model/Target	CAP Parameters	Drug/Agent	Effects	Ref.
Drug delivery	In vitro HeLa monolayer model	He, 0.5 L/min (DBD-based APPJ)	Propidium iodide and FITC-Dextran	Increased cell permeability and drug uptake	[98]
Drug delivery	In vitro blood–brain barrier model	Ar, NF (APPJ) Air (DBD)	Fluorescein isothiocyanate dextran	Increased cell permeability and drug uptake	[99]
Surface functionalisation for drug delivery	Micromotor delivery medium (acidic buffer)	Ar, 4–5 L/min (Hybrid DBD-APPJ)	Doxorubicin loaded gold-hyaluronic acid tubular micromotors	Increased drug release	[101]
Drug delivery and skin rejuvenation	Dorsal skin of male Wistar rats	Air (DBD)	Vitamin C	Increased collagen synthesis and epidermal thickness	[100]
Drug delivery and wound healing	Dermal wounds of diabetic New Zealand white rabbits	He, 5 L/min (Hybrid DBD-APPJ)	Collagen	Enhanced wound closure, angiogenesis, and reduced inflammation	[30]

Ar: Argon, APPJ: Atmospheric pressure plasma jet, DBD: Dielectric barrier discharges, He: Helium.

## Data Availability

No new data were created or analyzed in this study. Data sharing is not applicable to this article.

## Appendix A

**Table A1:** In vitro studies demonstrating therapeutic effects of CAP treatment towards cellular inflammatory responses, tissue repair processes and oxidative stress.

Cell Type	Stimulus	Gas	Type of Device	Flow Rate (L/min)	Time (s)	Reported Effects	Translational Relevance to OA	Ref.
Human monocytic cell line, THP-1	LPS	N	APPJ	1	20, 40, 70, 150	Reduced inflammatory responses: ↓ Gene expression of IL-6, IL-1β, IL-23, CXCL8, and MHC class II-related genes, ↓ IL-6 protein levels. Activation of antioxidant pathways: ↑ Expression of NRF2-regulated genes.	Non-OA-specific cell source	[85]
Human keratinocytes, HaCaT	TNF-α, IFN-γ	Air	DBD	NF	300, 600	Reduced inflammatory responses: ↓ Gene expression of IL1B, IL6, IL8, CCL17, CCL22 and protein levels of IL-6, TNF-α, CCL17.	Non-OA-specific cell source	[86]
Human gingival fibroblasts, HGF-1	Erastin	He and O	DBD	4 and 0.05	10, 30, 60, 120, 180	Reduced inflammatory responses: ↓ Gene expression of iNOS, COX-2, IL-1β, TNF-α, IL-6, and ↓ protein expression of iNOS and COX-2. Improved Mitochondrial function: ↑ VDAC1 expression, restored mitochondrial membrane potential and ↓ Mitochondrial ROS (MitoSOX).	Non-OA-specific cell source	[91]
Primary Human Rheumatoid Arthritis Fibroblast-Like Synoviocytes (RA-FLS)	TNF-α, LPS	Ar	APPJ	2.5	30, 45, 60, 90, 120	Reduced inflammatory responses: ↓ Gene expression of NF-κB (RelA) and protein levels of IL-6 and RANKL ↓ MMP-3 expression in unstimulated cells	Non-OA-specific cell source Inflammatory joint disease cell source	[83]
Primary human dermal fibroblasts	NT	Ar	Microwave-Induced Plasma Torch	4	120	Enhanced Pro-Healing Cytokine & Chemokine Production: ↑ Secretion of IL-6, IL-8, MCP-1 (CCL2), and Serpine E1 (PAI-1) and ↑ gene & protein levels of TGF-ß1 and TGF-ß2 Accelerated Fibroblast Migration: ↑ Migration rate in scratch wound assay Increased ECM & Myofibroblast Activation: ↑ Collagen type I and α-SMA gene expression	Non-OA-specific cell source	[95]
Primary human skin keratinocytes	NT	Ar	Microwave-Induced Plasma Torch	4	120	Increased wound healing responses: ↑ Expression of TGF-β1 and TGF-β2 in skin Anti-microbial activity: ↑ Gene expression of HBD-2, MBD-2, MBD-3	Non-OA-specific cell source	[92]
RA-FLS cultures (Origin not specified)	NT	Air	DBD	NF	60, 120	↑ Intracellular ROS content	Non-OA-specific cell source Inflammatory joint disease cell source	[80]
Primary Human OA Fibroblast-Like Synoviocytes	NT	Ar	APPJ	2.5	30, 45, 60, 90	Reduced inflammatory responses: ↓ Protein levels of IL-6	OA-specific cell source	[84]

APPJ: Atmospheric pressure plasma jet, Ar: Argon, CCL: Chemokine (C-C motif) Ligand, COX: Cyclooxygenase, CXCL: C-X-C Motif Chemokine Ligand, DBD: Dielectric barrier discharges, He: Helium, HBD: Human Beta-Defensin, IFN: Interferon, IL: Interleukin, iNOS: Inducible Nitric Oxide Synthase, L: litre, LPS: Lipopolysaccharide, MBD: Mouse Beta-Defensin, MHC: Major Histocompatibility Complex, MitoSOX: Mitochondrial ROS indicator, MMP: Matrix Metalloproteinase, N: Nitrogen, NF: No Flow, NF-κB: Nuclear Factor Kappa B, NRF2: Nuclear Factor Erythroid 2-Related Factor, NT: Not pre-stimulated with an inflammatory agent, O: Oxygen, OA: Osteoarthritis, PAI: Plasminogen Activator Inhibitor, RANKL: Receptor Activator of Nuclear Factor Kappa-B Ligand, s: seconds, SMA: Smooth Muscle Actin, TGF: Transforming Growth Factor, TNF: Tumour Necrosis Factor, VDAC: Voltage-Dependent Anion Channel, ↓: Decreased, ↑: Increased.

**Table A2:** Pre-clinical studies demonstrating therapeutic effects of CAP treatment towards inflammatory responses, tissue repair processes and oxidative stress in vivo.

Experimental Model	Species	Gas	Plasma Discharge Device	Flow Rate (L/min)	Exposure	Reported Effects	Translational Relevance to OA	Ref.
Adjuvant- induced rheumatoid arthritis (RA)	Male Sprague-Dawley rats	Air	APPJ	2 mL per joint	60 or 120 s, once every other day within 7 days. Joint cavity Exposure.	Reduced synovial hyperplasia, inflammation and vascular proliferation. Promoted antioxidant activity: ↑ GSH, SOD and CAT levels in synovial tissue	Non-OA-specific Inflammatory joint disease model CAP delivered into the knee joint cavity	[80]
Imiquimod- Induced Psoriasis-Like Skin Inflammation	Female BALB/c mice	Air, NF	DBD	NF	300 or 600 s per day for 7 consecutive days. Dorsal skin exposure.	Reduced inflammatory responses: ↓ Serum levels of IgG2a, MPO, IL-6 and TNF-α. ↓ Gene expression of IL-17A, IL-1β, IL-36, IL-6, and CXCL1 in skin.	Non-OA-specific Inflammatory skin disease model General CAP mechanistic data	[86]
Adenine- induced chronic kidney disease (CKD) and related skin disease	Male and female C57BL/6 mice	Air	DBD	NF	180 s twice daily for 14 consecutive days. Dorsal skin exposure.	Improved epidermal thickness and enhanced keratinocyte proliferation. Reduced inflammatory responses: ↓ IL-6 and TNF-α and inflammatory cell infiltration (CD3+, CD45+ and F4/80+) in skin.	Non-OA-specific CKD-related skin inflammation model General CAP mechanistic data	[87]
Streptozotocin (STZ) and nicotinamide-induced diabetes.	Male BALB/c mice	Ar	APPJ	3	600 s Dorsal skin exposure.	Reduced oxidative stress: ↓ Serum levels of MDA, AOPP, and oxLDL Reduced inflammatory responses: ↓ Serum levels of IL-1α, IL-1β, TNF-α and IL-6 Enhanced antioxidant activity: ↑ Glutathione peroxidase activity, ↓ H_2_O_2_.	Non-OA-specific General CAP mechanistic data	[88]
Dorsal skin injury in diabetic mice	Male db/db mice	He	APPJ	3	90 or 180 s for 14 consecutive days. Wound site exposure.	Accelerated wound healing: ↑ TGF-β and VEGF levels in skin. Reduced inflammatory responses: ↓ IL-6, TNF-α, iNOS and SOD levels in skin	Non-OA-specific Wound healing model General CAP mechanistic data	[93]
Wild-type mice	Female 129Sv/Ev mice	Ar	Microwave- Induced Plasma Torch	4	120 s for 5 days. Dorsal skin exposure.	Increased wound healing responses: ↑ Expression of TGF-β1 and TGF-β2 in skin Anti-microbial activity: ↑ levels of ß-defensins in skin.	Non-OA-specific General CAP mechanistic data	[92]
Induction of dorsal skin injury in wild-type mice	129Sv/Ev mice	Ar	Microwave- Induced Plasma Torch	4	120 s daily for 10 days. Wound site exposure.	Accelerated wound closure and tissue repair responses: ↑ Collagen type 1 expression. ↑ dermal MCP-1 and IL-6.	Non-OA-specific Wound healing model General CAP mechanistic data	[95]
STZ-induced diabetes with dorsal skin wounds	Male Sprague-Dawley rats	Ar	APPJ	5	120 s (small wounds) or 240 s (large wounds) daily until wound healing was complete.	Enhanced wound healing and collagen deposition. Increased antioxidant activity: ↑ Tissue levels of GSH, SOD and CAT	Non-OA-specific Diabetic wound healing model General CAP mechanistic data	[94]
Acute Surgical Incision and Revision Surgery Model	Sprague-Dawley rat	He	APPJ	4	30 s	Increased antioxidant activity: ↑ Catalase ↓ Apoptosis (GSEA pathway) Reduced inflammatory responses: ↓ IL-6, JAK/STAT Reduced fibrosis and adipogenesis	Non-OA-specific Muscle injury repair model General CAP mechanistic data	[89]

AOPP: Advanced Oxidation Protein Products, APPJ: Atmospheric pressure plasma jet, Ar: Argon, CAT: Catalase, DBD: Dielectric barrier discharges, GSH: Glutathione, H_2_O_2_: Hydrogen Peroxide, He: Helium, Ig: Immunoglobulin, IL: Interleukin, iNOS: Inducible Nitric Oxide Synthase, L: litre, MCP: Monocyte chemoattractant protein, MDA: Malondialdehyde, MPO: Myeloperoxidase, NF: No Flow, oxLDL: Oxidised Low-Density Lipoprotein, s: seconds, SOD: Superoxide Dismutase, TGF: Transforming Growth Factor, TNF: Tumour Necrosis Factor, VEGF: Vascular Endothelial Growth Factor, GSEA: Gene Set Enrichment Analysis, ↓: Decreased, ↑: Increased.
